# Antioxidative and anti-inflammatory effects of vitamin C on the liver of laying hens under chronic heat stress

**DOI:** 10.3389/fvets.2022.1052553

**Published:** 2022-10-28

**Authors:** Jun Du, Yan Shi, Changming Zhou, Lianying Guo, Ruiming Hu, Cheng Huang, Guoliang Hu, Xiaona Gao, Xiaoquan Guo

**Affiliations:** ^1^Jiangxi Provincial Key Laboratory for Animal Health, Institute of Animal Population Health, College of Animal Science and Technology, Jiangxi Agricultural University, Nanchang, China; ^2^School of Computer and Information Engineering, Jiangxi Agricultural University, Nanchang, China

**Keywords:** chronic heat stress, oxidative stress, inflammation, vitamin C, liver, laying hen

## Abstract

In this study, we investigated the therapeutic effect and mechanism of action of vitamin C on chronic heat stress (CHS)-induced liver oxidative damage and inflammation in laying hens. The thermoneutral control group (TN group) was kept at a constant temperature of 22 ± 1°C, while the chronic heat stress group (CHS group) and the vitamin C supplemented group (HSV group) were exposed to heat stress (HS) (36 ± 1°C, 8 h/d). The TN and HS groups were fed the basic diet at will, and the HSV group was supplemented with 300 mg/kg of vitamin C on top of the basic diet. The experimental results showed a significant improvement in body weight and feed intake in the HSV group compared to the HS group. A significantly lower pH and higher $\text{HCO}{}_{3}^{-}$ and PCO_2_ levels were observed in the HSV group compared to the CHS group. As laying hens were supplemented with vitamin C, serum malondialdehyde (MDA) level was declined, superoxide dismutase (SOD) and glutathione peroxidase (GSH-Px) activities were increased, and total antioxidant capacity (T-AOC) was increased. Further, CHS induced an increase in the expression of inflammation-related genes and a decrease in the expression of antioxidant-related genes. In contrast, the addition of vitamin C reversed the effects of CHS, resulting in an increase in the expression of antioxidant-related genes and a decrease in the expression of inflammation-related genes. In conclusion, vitamin C can effectively alleviate CHS-induced acid-base imbalance in body fluids of laying hens and the oxidative damage and inflammatory response caused to the liver. Therefore, vitamin C can be used clinically as an effective drug to alleviate chronic heat stress in laying hens. This experiment provides clinical evidence and theoretical basis for the use of vitamin C as an effective drug to alleviate chronic heat stress in laying hens.

## Introduction

Global warming has increased in this century due to anthropogenic greenhouse gas emissions, and all life on earth faces the threat of global warming, which is expected to increase in scope and severity in the coming decades (1, 2). When the temperature increase exceeds 1.5°C, it will exceed the limit of the poultry's (broilers and laying hens, etc.,) own thermoregulation, thus exposing the poultry to heat stress (3, 4). A variety of factors determine how heat stress affects organisms, including species, breed, age, genetic potential, physiological status, and nutrition, etc., (5). Poultry is more sensitive to high temperatures than other species due to their specific physiology, such as lack of sweat glands and feather coverings, and higher metabolic rate, which makes high temperatures have a significant impact on the health, physiology and performance of poultry (6). High temperature, as a major stressor for poultry, can induce endocrine and electrolyte disruptions, negatively affecting production performance, endocrine system, antioxidant, and anti-inflammatory capacity of poultry (7). This will lead to weight loss, reduced egg production, and even death of poultry. The impact of this on poultry rearing and production is global and brings huge financial losses to the farming industry (6). It is therefore important to study the damage mechanisms caused by heat stress on poultry and to determine how heat stress can be treated and prevented.

Heat stress causes endocrine and metabolic disorders in laying hens, resulting in an imbalance in the acid-base balance of body fluids, a decrease in the activity of antioxidant enzymes such as SOD and GSH-Px, and an increase in MDA content (8, 9). As well as leading to a decrease in the expression level of antioxidant-related genes, the antioxidant capacity of the body is reduced, leading to oxidative stress (10). To confront oxidative damage, antioxidant enzymes in the antioxidant system play an irreplaceable role, of which heme oxygenase-1 (HO-1), glutathione sulfotransferase (GST) and superoxide dismutase 2 (SOD2) are the key antioxidant enzymes in the body antioxidant system (11, 12). Recent studies have shown that the development and persistence of inflammation are largely dependent on oxidative stress, and that oxidative damage leads to cellular metabolic disorders and dysfunction, resulting in inflammation (13). The effects of heat stress on birds are known to include causing damage to defense mechanisms and relative immunosuppression (14). However, the mechanism by which high ambient temperatures may play an immunosuppressive role is not fully understood (15). Inflammation is a defensive response of the organism to damage produced by various stimuli (16). Nuclear factor-κB (NF-κB) is a pro-inflammatory transcription factor that regulates inflammation and is induced by damage caused by oxidative stress. NF-κB can regulate the expression of pro-inflammatory cytokines such as interleukin-6 (IL-6), interleukin-8 (IL-8), and tumor necrosis factor-α (TNF-α) (17, 18). The nuclear factor-κB (NF-κB) inflammatory pathway is activated when the body is exposed to oxidative damage, thereby regulating the expression of pro-inflammatory cytokines, leading to an inflammatory response and activation of the inhibitor-κB kinase (IKK) complex (19, 20).

Vitamin C (ascorbic acid) is a water-soluble vitamin and a vital antioxidant that is widely distributed in various tissues (21, 22). In general, most animals have the ability to synthesize vitamin C. Some species (such as humans and guinea pigs) are incapable of synthesizing vitamin C because their bodies lack L-gulonolactone lactone oxidase (23). The role of L-gulonolactone oxidase is to convert glucose to vitamin C in the last biosynthetic pathway, so this enzyme is required for the synthesis of vitamin C. Although vitamin C is naturally synthesized in most species, it is rapidly depleted in states of stress and disease, resulting in lower plasma ascorbic acid concentrations (24). In this state, the animal's body consumes vitamin C faster than its ability to synthesize it, causing oxidative damage to the organism (25, 26). Supplementation of vitamin C to heat-stressed animals results in more efficient heat exchange between the organism and its environment, as well as regulating oxygen consumption by modulating physiological and metabolic changes (27). Dietary supplementation with vitamin C can improve growth performance, alleviate stress metabolic symptoms, enhance immune function, and reduce mortality (28–30), but it is not entirely clear how vitamin C promotes the function of the antioxidant and immune systems (31, 32). Several existing studies have shown that vitamin C has an effect on the production of cell adhesion factors in phagocytes, inflammatory cytokines, lymphocytes, and monocytes (31, 32). In contrast to other drugs used to treat heat stress, ascorbic acid has a non-pressurizing, safer, and more practical efficacy for alleviating stress. In addition, vitamin C is inexpensive, easy to administer, easy to absorb, non-toxic to the body, has no withdrawal period, and has no after-effects of overdose (33). There is reasonable evidence and support for the use of vitamin C as an anti-stress drug (34). Notably, however, some studies on vitamin C-related stress-mimicking oxidative damage and inflammatory responses are controversial and some are inconclusive. However, the mechanisms of how vitamin C counteracts heat stress-induced disruption of oxidative defense systems and attenuates the inflammatory response in poultry remain unclear.

Several existing studies suggest that vitamin C appears to play a key role in mitigating the damage caused by heat stress to the antioxidant system and immune system, among others, in the animal organism (29, 35). However, most of the current research on heat stress in poultry has focused on poultry such as broilers and Japanese quail, while relatively little research has been done on egg-laying hens. It is worth mentioning that most studies on heat stress also focus on acute heat stress (most studies focus on 2 h to a maximum of 18 h), while studies on chronic heat stress are relatively few and not in-depth (9, 36). Thus, the purpose of this study was to examine the effect of vitamin C on chronically heat-stressed laying hens' livers from the perspective of antioxidant-related genes and inflammation-related genes expression.

## Materials and methods

### Animals and experimental design

The experimental animals in this study were 10-week-old Hy-line brown laying hens. These chickens were first placed in a constant temperature and humidity room with an environment of 22°C and 55 ± 5 % relative humidity for 14 days of adaptive rearing. After 14 days, 180 healthy and similar weight (1.10 ± 0.03 kg) 12-week-old Hy-line brown bred laying hens were screened and randomly divided into 3 groups, each group was set with 6 parallel replicates (*n* = 6), and each replicates contained 10 chickens. A total of two constant temperature and humidity animal rooms are set up, and the temperature and humidity in the room are strictly controlled by a combination of an automatic temperature control system and a humidifier. Constant temperature and humidity room I: heat stress group, *ad libitum* basal with diet; heat stress + vitamin C treatment group, *ad libitum* basal with diet + 300 mg/Kg vitamin C. In the formal experimental stage of this chicken house, the temperature is controlled at 36 ± 1°C and the relative humidity is 55 ± 5 % from 9:00 to 18:00 every day, and the temperature is controlled at 22 ± 1°C and the relative humidity is 55 ± 5 % during the rest of the time. Constant temperature and humidity room II: the thermoneutral control group, *ad libitum* basis and diet. The chicken house is controlled at a temperature of 22 ± 1°C and relative humidity of 55 ± 5 % 24 h a day. The whole experiment took 12 h of illumination, and the experimental period lasted for 21 days.

### Sample collection

On days 7, 14, and 21 after the start of the formal experiment, the weight data of chickens in each group were weighed and recorded. Eight chickens were randomly selected from each experimental group, and blood was collected from the wing vein and then part of the blood sample was put into a syringe containing sodium heparin. The blood gas index was tested within 5 min, and the remaining blood sample was transferred to a vacuum blood collection tube without anticoagulant. Blood samples were centrifuged at 2,500 rpm for 15 min at 4°C to separate serums, which were then stored at−80°C until the test was performed. Laying hens were dislocated at the cervical region, liver samples were collected, immediately frozen in liquid nitrogen, and stored at−80°C for further study.

### Measurements of blood gas parameters

Use a 2.5 mL syringe to draw approximately 1.5 mL of blood sample, mix the blood sample by gentle manual rotation and inversion of the syringe, and use a blood gas analyzer for testing within 5 min of collection (37). i-STAT's test cards are stored in a refrigerator (4–8°C). Test cards should be warmed up at room temperature for 10 min before use. The i-STAT blood gas analyzer was calibrated and used according to the manufacturer's recommendations.

### Measurements of redox indicators

Serum malondialdehyde (MDA) levels and glutathione peroxidase (GSH-Px), superoxide dismutase (SOD), and total antioxidant capacity (T-AOC) activities were measured using commercial kits (Nanjing Jiancheng Institute of Biological Engineering, Nanjing, China). All experimental procedures were performed according to the manufacturer's recommended protocol.

### Analysis of real-time quantitative PCR (RT-QPCR)

RNA extraction, cDNA synthesis and q-PCR were performed with reference to the methods in previous studies (38, 39). According to the instructions of the total RNA extraction kit, the total RNA in liver tissue was extracted by Trizol method. The total RNA purity was calculated after the A260/A280 ratio was determined, and the whole experiment was performed on ice. According to the instructions in the commercial kit (Takara, Beijing China), 1 μg of total RNA was reverse transcribed into cDNA.

The forward (F) and reverse (R) primers of the detected genes are shown in Supplementary Table S1, synthesized by Beijing Tsingke Biotechnology Co., Ltd., (Changsha). Real-time RT-PCR reactions were performed on CFX96 Touch Real-time PCR Detect System (BIO-RAD, California, United States) using the SYBR Green method (Takara., Beijing China) as follows: 95°C, 2 min; 95°C, 30 s; 60°C, 30 s; 72°C, 30 s; a total of 40 cycles were run. Statistical analysis was performed using the 2^−ΔΔ^CT method with GAPDH as an internal control.

### Statistical analysis

We express data as means ± standard errors (SEM). Statistical analyses were performed using SPSS 17.0 software (SPSS Inc., Chicago, IL, United States) and plotted by GraphPad Prism 6.01 software (GraphPad Software, Inc., San Diego, CA). In order to compare the differences between multiple groups, we used least significant difference (LSD) tests and one-way analysis of variance (ANOVA). When *P* < 0.05, it indicates that the difference between the two data groups is significant, and when *P* < 0.01, it indicates that the difference between the two data groups is highly significant.

## Results

### Body weight and feed intake

**Figure 1:**
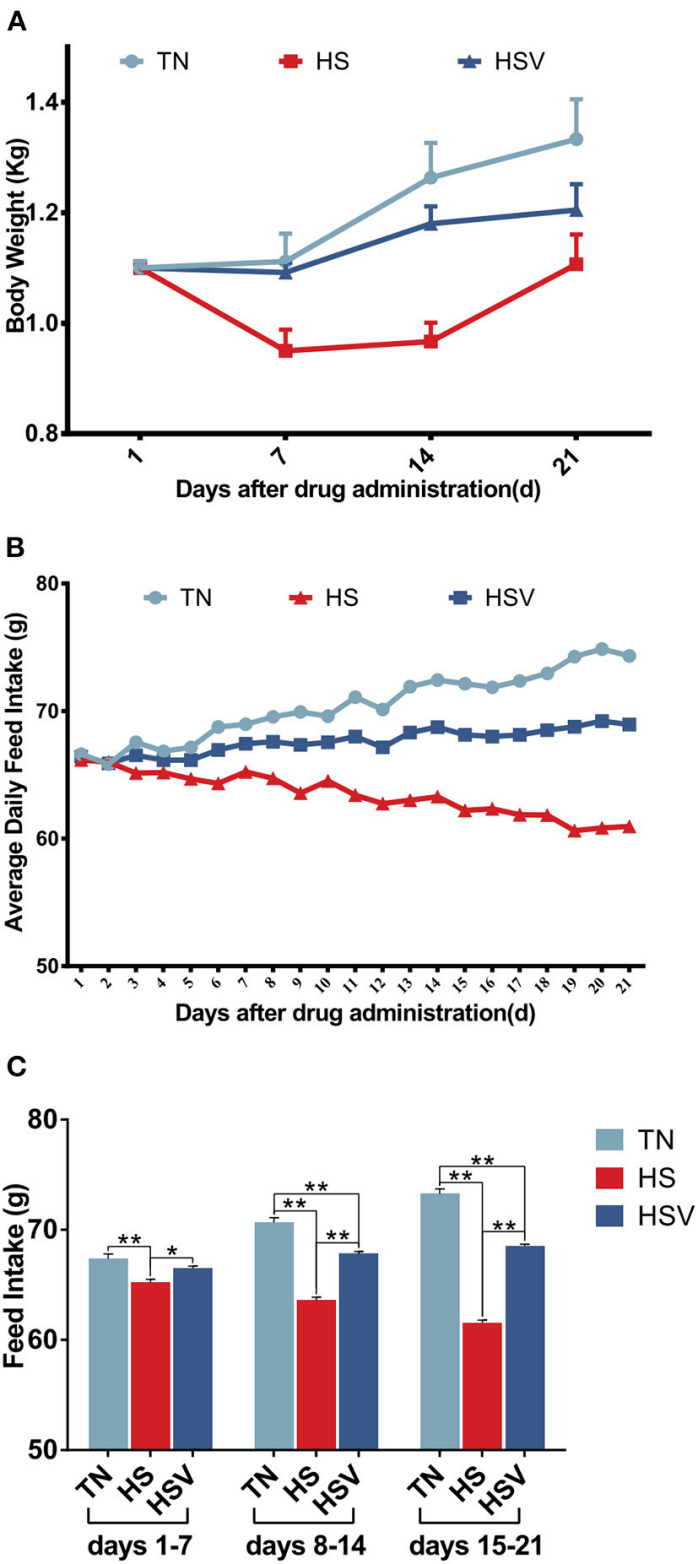
Effects of vitamin C on body weight and feed intake of chronically heat-stressed laying hens. **(A)** body weight; **(B)** average daily feed intake; and **(C)** feed intake. Data are expressed as mean ± SEM (*n* = 6). * indicates a significant difference (^*^P < 0.05 and ^**^P < 0.01). Same as below.

Figure 1 shows the body weight and feed intake trends. Body weight changes are shown in Figure 1A. Under chronic heat stress conditions, the body weight of laying hens in the HS group decreased significantly (*P* < 0.01) on days 7, 14, and 21 compared with the TN group; while the body weight of laying hens in the HSV group increased significantly (*P* < 0.01) on days 7 and 14 compared with the HS group. Figures 1B,C illustrate changes in feed intake. The feed intake of hens in the HS group on days 1–7, 8–14, and 15–21 was significantly lower than that in the TN group *(P* < 0.01); whereas the feed intake of hens in the HSV group on days 1–7, 8–14, and 15–21 was significantly higher than that in the HS group (*P* < 0.05 or *P* < 0.01).

### Blood chemistry parameters

**Figure 2:**
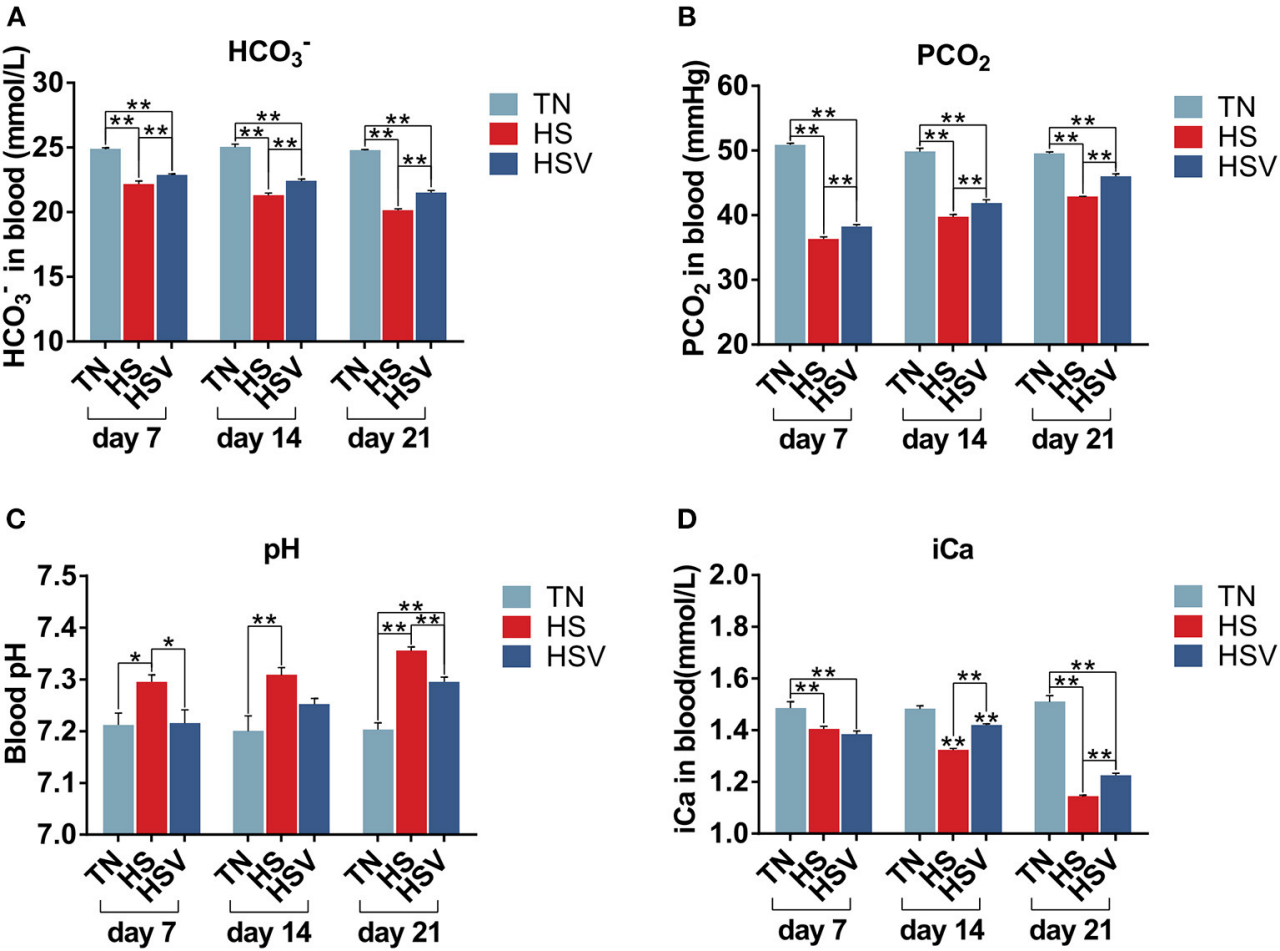
Effects of vitamin C on blood gas indexes in chronically heat-stressed laying hens. **(A)** blood chemistry parameters of HCO${}_{3}^{-}$; **(B)** blood chemistry parameters of PCO_2_; **(C)** blood chemistry parameters of pH; and **(D)** blood chemistry parameters of iCa.

Figure 2 illustrates vitamin C's effects on chronically heat-stressed hen blood gas concentrations. Blood pH, $\text{HCO}{}_{3}^{-}$, PCO_2_, and iCa levels were significantly lower (*P* < 0.05 or *P* < 0.01) in the HS group of hens compared to the TN group on days 7, 14, and 21. Compared with the HS group, hens in the HSV group had significantly lower blood pH on days 7 and 21 (*P* < 0.05 or *P* < 0.01); significantly higher blood $\text{HCO}{}_{3}^{-}$ and PCO_2_ levels on days 7, 14, and 21 (*P* < 0.01); and significantly higher blood iCa levels on days 14 and 21 (*P* < 0.01).

### Antioxidant index

**Figure 3:**
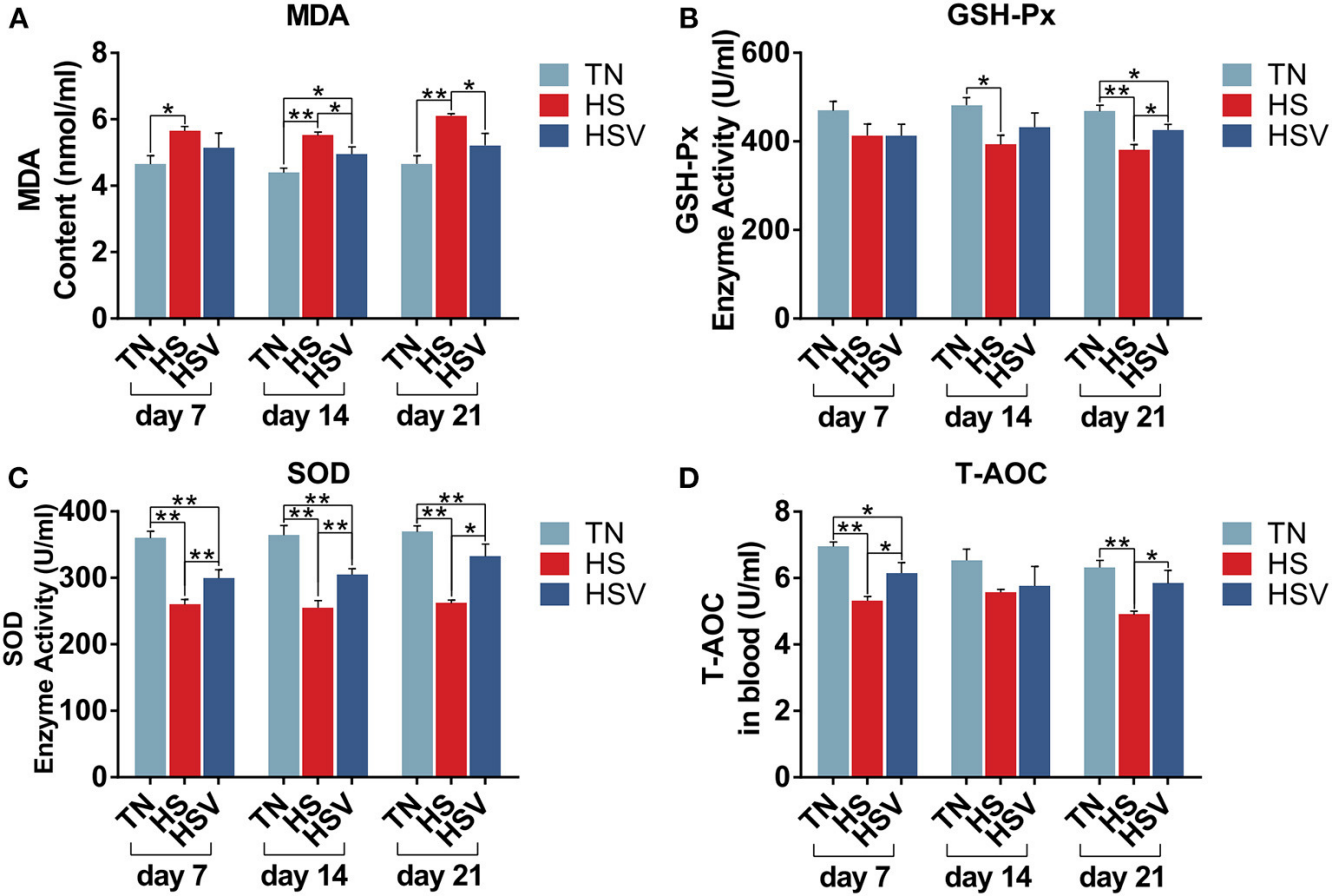
Effects of vitamin C on liver redox status of chronically heat-stressed laying hens. **(A)** blood chemistry parameters of MDA; **(B)** blood chemistry parameters of GSH-Px; **(C)** blood chemistry parameters of SOD; and **(D)** blood chemistry parameters of T-AOC.

The antioxidant levels of each group on days 7, 14, and 21 are shown in Figure 3. Compared with the TN group, serum malondialdehyde (MDA) levels were significantly higher in the HS group on days 7, 14, and 21 (*P* < 0.05 or *P* < 0.01); serum superoxide dismutase (SOD), glutathione peroxidase (GSH-Px) activities and serum total antioxidant capacity (T-AOC) levels were significantly lower on days 7, 14, and 21 (*P* < 0.05 or *P* < 0.01). Serum superoxide dismutase (SOD) levels, serum total antioxidant capacity (T-AOC) levels and serum peroxidase (GSH-Px) levels on days 7, 14, and 21 were significantly increased in the HSV group compared to the HS group (*P* < 0.05 or *P* < 0.01).

### Effects of vitamin C on chronic heat stress-induced changes in the mRNA expression of oxidative stress genes in the liver

**Figure 4:**
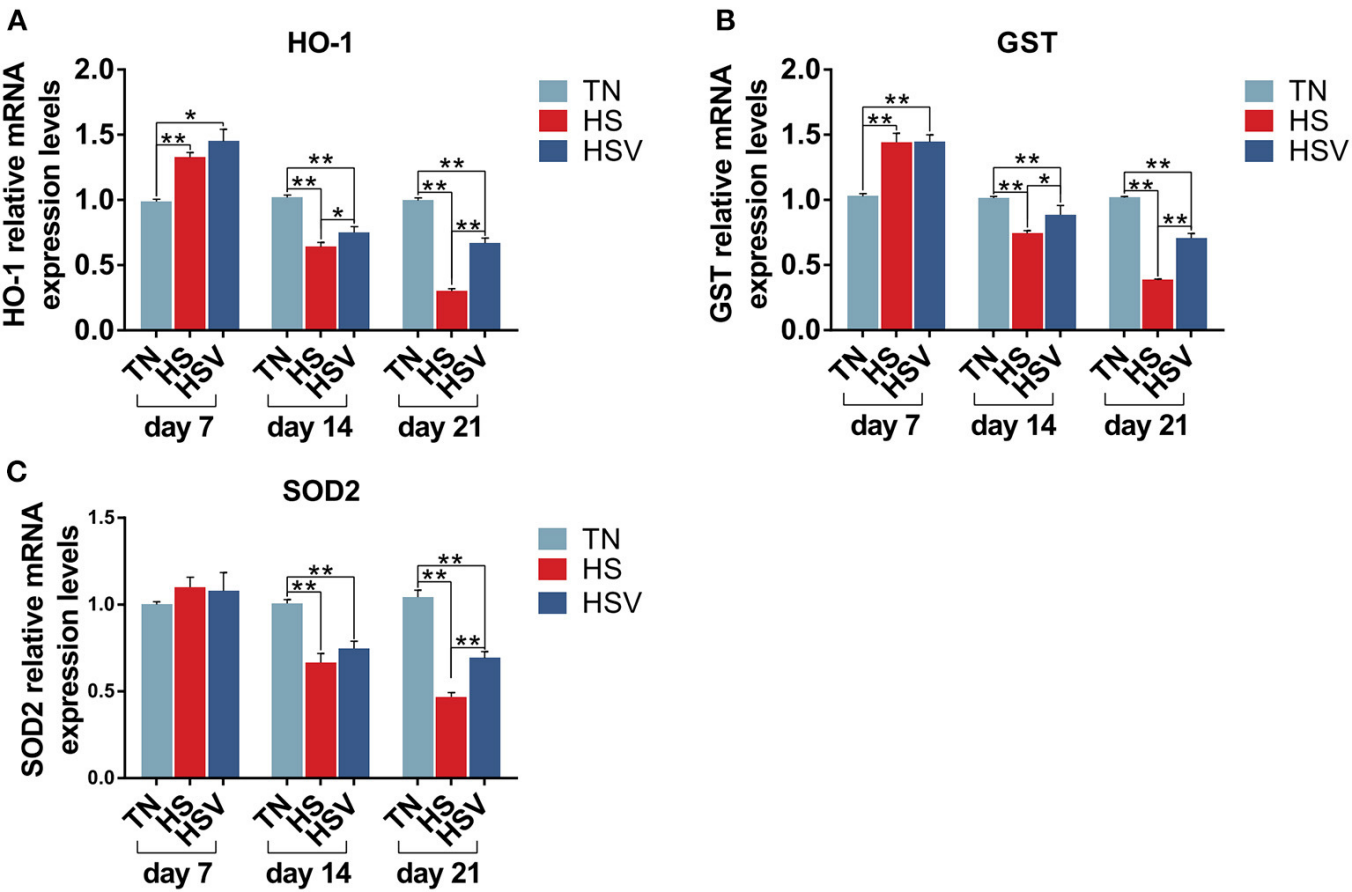
Effects of vitamin C on chronic heat stress-induced changes in the mRNA expression of oxidative stress genes in the liver. **(A)** mRNA expression of HO-1; **(B)** mRNA expression of GST; and **(C)** mRNA expression of SOD2.

Figure 4 shows the expression of antioxidant genes in the liver of laying hens. The mRNA expression levels of HO-1 and GST in the liver of hens in the HS group were significantly higher on day 7 compared with the TN group (*P* < 0.01); the mRNA expression levels of HO-1, GST, and SOD in the liver were significantly lower on days 14 and 21 compared with the TN group. HO-1 and GST expression levels in the liver of hens in the HSV group on days 14 and 21 and SOD2 expression levels in the liver on day 21 were significantly lower. On days 14 and 21, the liver mRNA expression levels of HO-1, GST, and SOD2 were significantly lower in the HSV group than in the HS group (*P*<0.05 or *P*<0.01).

### Effects of vitamin C on chronic heat stress-induced changes in the mRNA expression of inflammatory genes in the liver

**Figure 5:**
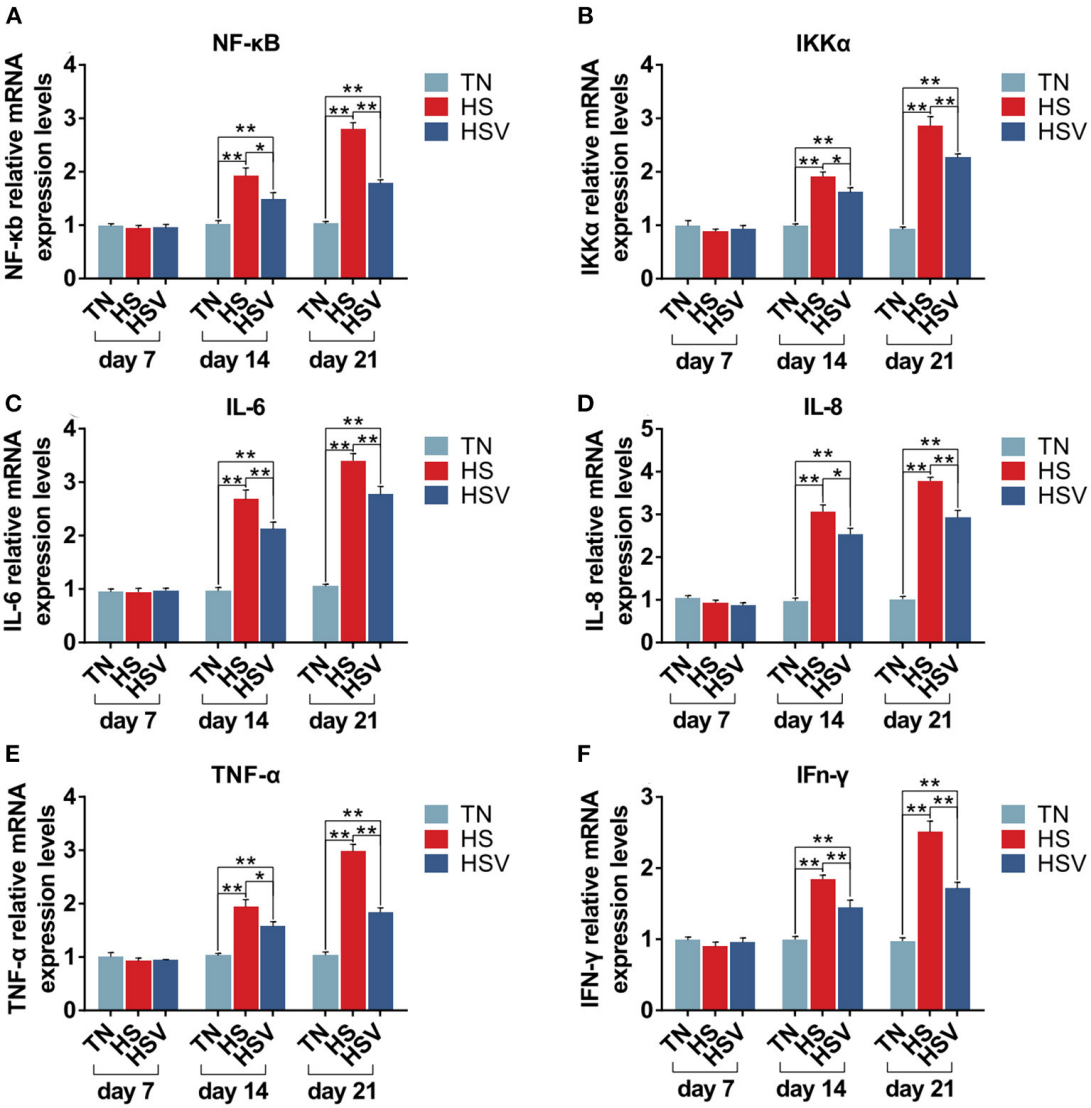
Effects of vitamin C on chronic heat stress-induced changes in the mRNA expression of inflammatory genes in the liver. **(A)** mRNA expression of NF-κB; **(B)** mRNA expression of IKK-α; **(C)** mRNA expression of IL-6; **(D)** mRNA expression of IL-8; **(E)** mRNA expression of TNF-α; and **(F)** mRNA expression of IFn-γ.

Figure 5 illustrates the expression of inflammation-related genes in the liver of laying hens. Compared with the TN group, the mRNA expressions of NF-κB, IKK-α, IL-6, TNF-α, IL-8, and IFN-γ in the liver of laying hens in the HS group were significantly decreased on the 14th and 21st days *(P*<0.05 or *P*<0.01). The mRNA expression levels of NF-κB, IKK-α, IL-6, TNF-α, IL-8, and IFN-γ in the liver of laying hens in the HSV group were significantly lower than those in the HS group on the 14th and 21st days (*P*< 0.05 or *P*<0.01).

## Discussion

Available studies have shown that heat stress negatively affects the productive performance of poultry as well as physiological and biochemical indicators, which can lead to oxidative damage and inflammation in severe cases (40, 41). However, the mechanism of long-term heat stress on the liver injury of laying hens is still unclear. Meanwhile, existing studies have demonstrated the alleviating effects of some drugs on stress, especially heat stress, but there is a general lack of systematic and clinical studies on the mechanisms and methods by which these drugs prevent and alleviate chronic heat stress (42, 43). Our study investigated whether vitamin C could alleviate heat stress-induced liver injury in laying hens and the mechanism of action of vitamin C to alleviate liver injury. As a result of this study, vitamin C had significant therapeutic effects on the liver of laying hens and was able to relieve oxidative stress and inflammation caused by heat stress.

Compared to other species, poultry have a special physiological structure, the air sac, which plays a crucial role in gas exchange (6, 44). It is worth noting that under conditions of chronic heat stress, enhanced respiration leads to a decrease in the partial pressure of carbon dioxide in the blood, a decrease in the concentration of bicarbonate ions and an increase in blood pH (respiratory alkalosis), which hinders the supply of bicarbonate required for eggshell mineralization and leads to an increase in the supply of organic acids and also decreases the level of free calcium in the blood (45). The results showed that high temperature decreased plasma carbon dioxide partial pressure, bicarbonate, free calcium content, and increased plasma pH in the HS group. Our findings were the same as the previous studies, except that we measured a significant increase in pH in the HS group, but it did not lead to alkalosis in the body (28, 45). In response to this result, we analyzed that it could be that birds under long-term cyclic heat stress go through a respiratory cycle like the wheezing and non-wheezing periods, which cause only temporary alkalosis (only during the panting period) in a high-temperature environment, resulting in a dramatic change in blood pH in laying hens (46). Therefore, the time of blood collection may be important for the detection of pH. In contrast, when we collected blood from birds, they were not under heat stress, so pH was significantly higher in the CHS group than in the TN group without causing alkalosis. The HSV group had significantly improved blood gas indices compared with the HS group, suggesting that vitamin C can significantly improve acid-base disturbances caused by heat stress.

The results of antioxidant indexes showed that serum MDA levels increased, SOD and GSH-Px activities decreased, and T-AOC levels decreased in the HS group. Meanwhile, the expression levels of antioxidant genes HO-1, SOD2 and GSH in the liver of the HS group were significantly decreased on days 14 and 21. This result supports the idea that oxidative stress is part of the response to heat stress in poultry (9) and is consistent with the experimental results obtained by Liao et al. (11) and Gao et al. (47). Available studies have shown that heat stress can induce oxidative stress (OS) (7) and that heat stress is an important stressor of oxidative stress, while the liver is the main organ under attack (48). Meanwhile, our results showed that vitamin C significantly attenuated heat stress-induced hepatic oxidative stress on days 14 and 21, which may be responsible for the increase in body weight and average daily feed intake of laying hens. Similar findings were found by Mossler, et al. (35), who also indicated that vitamin C could mitigate oxidative stress. Although laying hens themselves can synthesize vitamin C, in certain environments, such as in a heat-stressed environment, the metabolic demands of the organism can exceed its synthetic capacity (35). The supplementation of vitamin C to heat-stressed broilers has been shown to positively affect their health and growth performance. For example, the addition of 250 mg/kg of vitamin C to broilers reared for 42 days in a heat-stressed environment (32°C) resulted in a significant increase in live weight, feed efficiency, and carcass characteristics, as well as a reduction in MDA levels (49). In addition to this, the role of vitamin C in regulating the antioxidant capacity of monogastric animals (50), ruminants (34, 51, 52), and even humans (53) under stress stimulation are well–documented. However, we also observed a phenomenon that the expression levels of antioxidant genes HO-1, SOD2, and GSH were increased in the liver of the HS group on day 7 of the experiment, even higher than in the TN group. This may be because in the early stage of heat exposure, the antioxidant defense mechanism of laying hens is not damaged, and they are still trying their best to prevent oxidative damage and maintain the redox state balance in the body.

It is well known that oxidative stress can damage the body's antioxidant and immune systems and trigger an inflammatory response (54, 55). Inflammation and oxidative stress interact to cause a series of reactions called the oxidative inflammatory cascade, in which oxidative damage exacerbates the body's inflammatory response (54, 55). In an article by Jin et al. (56), heat stress was shown to activate NF-κB and spur the release of inflammatory factors (IL-6, IL-8, TNF-α) that can damage and necrosis organisms (56, 57). NF-κB is an important regulatory factor in the body (58). It participates in the body's inflammatory response, immune response, and oxidative tissue damage, and plays a vital role in maintaining the normal physiological function of cells (59). In many cases, NF-κB activation is associated with the synthesis of proinflammatory cytokines (60). In addition, phosphorylation of IKK leads to NF-κB activation through IκBα ubiquitination and proteasomal degradation in response to heat stress (61). Existing research has demonstrated that IFN-γ's synergistic effect is unrelated to the activation of transcription factors like NF-κB (62). The experimental results showed that the expression of IKK-α, NF-κB, IL-6, IL-8, TNF-α, and IFN-γ genes were higher in the HS group than those in the TN group on 14 and 21 days. Moreover, the expression levels of the above-mentioned genes were significantly decreased in the HSV group compared with the HS group. This study suggests that heat stress can activate NF-κB, and can promote the mRNA of IL-6, IL-8, and TNF-α mRNA, and increase the expression levels of IKK-α and IFN-γ gene mRNA, while in the diet the addition of vitamin C to heat-stressed laying hens alleviated the inflammatory response. However, we analyzed the test results and found that there were no significant differences in the expression levels of different genes on day 7, so we made a reasonable inference by combining the expression levels of antioxidant genes on day 7. Since the antioxidant system and antioxidant genes were not yet damaged by heat stress on day 7, the antioxidant system of laying hens still played a role in enhancing the body's ability to resist heat stress. At this time, the immune system is not yet affected and the body is not yet experiencing an inflammatory response. In the middle and late stages of heat stress (days 14 and 21), the continuous heat stress leads to the disruption of the body's antioxidant system, which in turn leads to an inflammatory response.

Chronic heat stress causes an imbalance in acid-base balance in Hy-line brown laying hens and negatively affects body weight and average daily feed intake. As a result, serum malondialdehyde levels increased significantly, antioxidant enzymes (SOD, GSH-Px) and T-AOC content decreased, and the expression of antioxidant genes (HO-1, SOD2, GSH) in liver tissue was also significantly decreased. Under the influence of oxidative stress, the expression of NF-κB was activated, as was the expression of IKK-α, IFN-γ and downstream inflammatory cytokines (IL-6, IL-8, TNF-α), leading to an inflammatory response *in vivo*. In contrast, the addition of vitamin C to the diet was effective in improving the disturbance of acid-base balance, alleviating the effects of heat stress on body weight and average daily feed intake, significantly reducing malondialdehyde content, increasing the activity of antioxidant enzymes inhibited by heat stress, increasing the expression levels of antioxidant genes (HO-1, SOD2, and GSH), and reducing the expression levels of inflammation-related genes (NF-κB, IKK-α, IFN-γ, IL-6 IL-8, TNF-α) expression levels. Thus, we conclude that vitamin C can improve the disturbance of acid-base balance in laying hens caused by chronic heat stress and alleviate the oxidative stress response of the body, thus reducing the inflammatory response of the body.

## Conclusion

This experiment clearly showed that chronic heat stress reduced the growth rate and feed intake of laying hens, resulting in elevated blood pH and causing oxidative damage and inflammation in the liver of laying hens. Adding vitamin C to the feed alleviated the liver damage caused by chronic heat stress. Vitamin C improved the body's resistance to oxidative stress and anti-inflammatory capacity by regulating the expression of antioxidant genes HO-1, GSH, SOD2 and inflammation-related genes NF-κB, IKKα, IL-6, TNFα, IL-8, IFN-γ at different time points. In addition, vitamin C can maintain the pH balance in CHS-laying hens by regulating blood pH.

## Data availability statement

The original contributions presented in the study are included in the article/Supplementary Materials, further inquiries can be directed to the corresponding authors.

## Ethics statement

The animal study was reviewed and approved by the Animal Ethics Committee of Jiangxi Agricultural University (Approval ID: license number JXAULL-2020-28).

## Author contributions

JD: data management, research, methodology, and writing manuscript. YS: data analysis, investigation, method, and visualization. CZ and LG: verification. RH: project management. CH: analysis. GH: software. XGa: correction and editor. XGu: conceptualization, funding access, methods, and supervision. All authors contributed to the article and approved the submitted version.

## Funding

This work was supported by the National Natural Science Foundation of China, Beijing, China Grants (32060760 and 31460679), the Natural Science Foundation of Jiangxi Province Grant (2017ACB20012), the Technology R&D Program of Jiangxi Province, Nachang, China Grant (GJJ210415), and Technology System of Modern Agricultural Poultry Industry of Jiangxi Province (JXARS).

## Conflict of interest

The authors declare that the research was conducted in the absence of any commercial or financial relationships that could be construed as a potential conflict of interest.

The handling editor AK declared a past co-authorship with the authors XGu and GH.

## Publisher's note

All claims expressed in this article are solely those of the authors and do not necessarily represent those of their affiliated organizations, or those of the publisher, the editors and the reviewers. Any product that may be evaluated in this article, or claim that may be made by its manufacturer, is not guaranteed or endorsed by the publisher.
