# Supplementary material for: Antioxidative and anti-inflammatory effects of vitamin C on the liver of laying hens under chronic heat stress
Source: Front Vet Sci. 2022 Oct 28;9:1052553. doi: 10.3389/fvets.2022.1052553 (PMC9650548; doi:10.3389/fvets.2022.1052553)
Supplement: Supplementary file 5 [file Data_Sheet_3.docx]

**Supplementary Information**

**Antioxidative and anti-inflammatory effects of vitamin C on the liver of laying hens under chronic heat stress**

**Jun Du^1†^, Yan Shi^2†^, Changming Zhou^1^, Lianying Guo^1^, Ping Liu^1^, Ruiming Hu^1^, Cheng Huang^1^, Guoliang Hu^1^, Xiaona Gao^1, *^ and** **Xiaoquan Guo^1, *^**

^1^Jiangxi Provincial Key Laboratory for Animal Health, Institute of Animal Population Health, College of Animal Science and Technology, Jiangxi Agricultural University, Nanchang, Jiangxi, China

^2^School of Computer and Information Engineering, Jiangxi Agricultural University, Nanchang, Jiangxi, China

†Jun Du and Yan Shi are equal first authors.

^*^Xiaoquan Guo and Xiaona Gao are the corresponding authors.

E-mail address: xqguo20720@jxau.edu.cn, xiaona.gao@jxau.edu.cn.

Table S1 Sequence of the primers used in real-time quantitative (RT-qPCR)

| RNA target | Accession no. | Primers sequences (5′–3′) | Product size(bp) |
| --- | --- | --- | --- |
| GAPDH | NM_204305 | F: 5’- TGGCATCCAAGGAGTGAGC -3’  R: 5’- GGGGAGACAGAAGGGAACAG -3’ | 141 |
| HO-1 | NM_205344.1 | F: 5’- GCTGAAGAAAATCGCCCAA -3’  R: 5’- ATCTCAAGGGCATTCATTCGG -3’ | 135 |
| GST | NM_001001776.1 | F: 5’- GGAAGCCATTTTAATGACAGA -3’  R: 5’- TCCTTTAAAAGCCTGTAGCAGA -3’ | 76 |
| SOD2 | XM_015285700.2 | F: 5’- TTTTCTCCTAAAGATGGCAAG -3’  R: 5’- CTTCCTGCTCATGGATCACAA -3’ | 109 |
| NF-κB | NM_205134.1 | F: 5’- GAAGGAATCGTACCGGGAACA -3’  R: 5’- CTCAGAGGGCCTTGTGACAGTAA -3’ | 131 |
| TNFα | AY765397 | F: 5’- TGTGTATGTGCAGCAACCCGTAGT -3’  R: 5’- GGCATTGCAATTTGGACAGAAGT -3’ | 229 |
| IKK-α | NM_001012904.1 | F: 5’- TTCACTGGTAAGCTCCAGCC -3’  R: 5’- TTCTCTTGCCTCCTGCAACA -3’ | 199 |
| IL-6 | HM179640 | F: 5’- AAATCCCTCCTCGCCAATCT -3’  R: 5’- CCCTCACGGTCTTCTCCATAAA -3’ | 106 |
| IL-8 | AJ009800 | F: 5’- GGCTTGCTAGGGGAAATGA -3’  R: 5’- AGCTGACTCTGACTAGGAAACTGT -3’ | 200 |
| IFN-γ | NM_205149 | F: 5’- ACTGAGCCAGATTGTTTCGAT -3’  R: 5’- TCTTTCACCTTCTTCACGCCAT -3’ | 135 |

Table S2 Experimental design

| Group | Temperature (℃) | Feed | Humidity (%) |
| --- | --- | --- | --- |
| TN | 22 ± 1 | basal diet | 55 ± 5 |
| HS | 36 ± 1 | basal diet + 300 mg/Kg Vc |  |
| HSV | 36 ± 1 | basal diet + 300 mg/Kg Vc |  |
